# Colon cancer in a patient with a mosaic monoallelic germline pathogenic *NF1* gene variant

**DOI:** 10.37349/en.2026.1006148

**Published:** 2026-09-03

**Authors:** Feras Alsabagh, Karam M. Chaaban, Marlene Girardo, Ehab Harahsheh, Misha B. Asif, Dusica Babovic-Vuksanovic, Mohamad Bassam Sonbol, Mayowa A. Osundiji

**Affiliations:** 1 CLS Health Comprehensive Care Clinic—Clear Lake, Webster, TX 77598, USA; 2 Department of Clinical Genomics, Mayo Clinic, Phoenix, AZ 85054, USA; 3 Department of Health Sciences Research, Mayo Clinic Arizona, Scottsdale, AZ 85259, USA; 4 Department of Neurology, Mayo Clinic, Phoenix, AZ 85054, USA; 5 Department of Clinical Genomics, Mayo Clinic, Rochester, MN 55905, USA; 6 Division of Medical Oncology, Mayo Clinic Cancer Center, Mayo Clinic, Phoenix, AZ 85054, USA

**Keywords:** *NF1*, colon, cancer, frameshift, germline

## Abstract

Pathogenic variants in the tumor suppressor gene *NF1* cause neurofibromatosis type 1 (NF1), one of the most common hereditary cancer predisposition syndromes. Pathogenic *NF1* variants have been associated with an increased risk of several cancers; however, the relationship between *NF1* variation and colon cancer remains underreported. We report a 41-year-old woman with a mosaic monoallelic germline pathogenic *NF1* variant, NM_000267.3:c.1756_1759del (p.Thr586Valfs*18), previously detected on germline multigene panel testing in saliva at a variant allele frequency of approximately 35%. She later presented with fatigue, dyspnea on exertion, and iron-deficiency anemia. Computed tomography, colonoscopy, biopsy, mismatch repair immunohistochemistry, surgical pathology, and tumor next-generation sequencing led to the diagnosis of right-sided colon adenocarcinoma that was mismatch repair deficient (dMMR) and microsatellite instability-high (MSI-H). She underwent right hemicolectomy, recovered postoperatively, and entered standard surveillance; at the time of manuscript development, she was also receiving systemic therapy. This case highlights the co-occurrence of an *NF1* variant and dMMR colorectal cancer and underscores the need for further studies to determine whether this represents a coincidental finding or a biologically meaningful association.

## Introduction

Neurofibromatosis type 1 (NF1) is an autosomal dominant tumor predisposition syndrome caused by pathogenic variants in the *neurofibromin 1* (*NF1*) gene on chromosome 17q11.2 [1, 2]. NF1 affects approximately 1 in 2,500 to 1 in 3,000 individuals worldwide and is among the most common inherited cancer syndromes [3, 4]. Major manifestations include café-au-lait macules, axillary or inguinal freckling, cutaneous and plexiform neurofibromas, Lisch nodules, optic pathway glioma, skeletal abnormalities such as scoliosis, and learning or developmental difficulties. The *NF1* gene encodes neurofibromin, a negative regulator of the rat sarcoma (RAS) signaling pathway. The gene spans approximately 280–282 kb and includes 57 constitutive exons with alternatively spliced exons reported in some annotations [2]. Loss of neurofibromin function can upregulate downstream RAS/mitogen-activated protein kinase (MAPK) signaling and promote abnormal cell growth [5]. Pathogenic *NF1* variants have been associated with an increased risk of several neoplasms, including gliomas, malignant peripheral nerve sheath tumors, neurofibromas, pheochromocytomas, breast cancer, and gastrointestinal stromal tumors [1, 6–10]. Whether *NF1* variants are also associated with increased colon cancer susceptibility remains uncertain.

Colonic adenocarcinoma is a malignant epithelial neoplasm arising from gland-forming cells of the colonic mucosa. Colorectal cancer is the third most commonly diagnosed cancer worldwide and a leading cause of cancer-related mortality; in 2020 there were approximately 2 million new cases globally, and by 2040 the global burden is projected to increase substantially [11]. In the United States, an estimated 152,810 new colorectal cancer cases and 53,010 deaths were expected in 2024 [12]. Common signs and symptoms include iron-deficiency anemia, fatigue, change in bowel habits, rectal bleeding, abdominal pain, weight loss, and bowel obstruction; diagnosis is typically established using colonoscopy with biopsy, cross-sectional imaging, and pathologic staging [13]. Universal mismatch repair testing is recommended in newly diagnosed colorectal cancer because deficient mismatch repair (dMMR) and microsatellite instability-high (MSI-H) status have diagnostic, prognostic, and therapeutic implications [14, 15]. Only a small number of reports have linked germline pathogenic *NF1* variants to colorectal malignancy [16, 17]. This limited literature leaves an important research gap regarding whether specific *NF1* variants, particularly mosaic loss-of-function variants, may contribute to noncanonical gastrointestinal tumor risk. Here, we report a case of invasive colonic adenocarcinoma in a 41-year-old woman of Hispanic descent with a mosaic monoallelic germline pathogenic *NF1* variant, NM_000267.3:c.1756_1759del (p.Thr586Valfs*18), further supporting the need for systematic study of possible colorectal cancer risk in NF1.

## Timeline

A chronological summary of the patient’s major clinical events, including the mosaic NF1 diagnosis and associated complications, is presented in Figure 1. The patient’s clinical course, including germline testing, diagnostic evaluation, definitive surgical treatment, and postoperative management, is summarized in Table 1.

## Narrative

CARE case report guidelines were followed in preparing this manuscript [18]. This study was deemed exempt from review by the Mayo Clinic Institutional Review Board, and informed consent was obtained from the patient for publication. A 41-year-old woman of Hispanic descent with a mosaic monoallelic germline pathogenic *NF1* variant, NM_000267.3:c.1756_1759del (p.Thr586Valfs*18), later presented for outpatient evaluation of fatigue, dyspnea on exertion, and anemia that prompted workup for gastrointestinal blood loss and subsequent diagnosis of colon cancer. The germline variant had been detected approximately 2 years earlier in a non-malignant saliva sample during multigene hereditary cancer panel testing performed in the context of hearing loss, scoliosis, and developmental delay. The variant allele frequency (VAF) was approximately 35%, consistent with post-zygotic mosaicism. The multigene panel was performed by Invitae Laboratories (San Francisco, CA, USA) with an average sequencing depth of approximately 300× to 500× and variant interpretation using the SHERLOC framework [19]. The panel included *APC*, *ATM*, *AXIN2*, *BARD1*, *BMPR1A*, *BRCA1*, *BRCA2*, *BRIP1*, *CDH1*, *CDK4*, *CDKN2A*, *CHEK2*, *CTNNA1*, *DICER1*, *EPCAM*, *GREM1*, *HOXB13*, *KIT*, *MEN1*, mutL homolog 1 (*MLH1*), mutS homolog 2 (*MSH2*), *MSH3*, *MSH6*, *MUTYH*, *NBN*, *NF1*, *NTHL1*, *PALB2*, *PDGFRA*, postmeiotic segregation increased 2 (*PMS2*), *POLD1*, *POLE*, *PTEN*, *RAD50*, *RAD51C*, *RAD51D*, *SDHA*, *SDHB*, *SDHC*, *SDHD*, *SMAD4*, *SMARCA4*, *STK11*, *TP53*, *TSC1*, *TSC2*, and *VHL*. No pathogenic Lynch syndrome-associated germline variant was identified. Relatives were unavailable for targeted familial testing of the *NF1* variant.

Family history was notable for colon cancer in the patient’s maternal grandfather in his 80s and breast cancer in the patient’s mother, who died in her early 60s in the context of COVID-19. No additional family history suggestive of Lynch syndrome or confirmed NF1 was reported. Historical features prompting germline evaluation included hearing loss, scoliosis, and developmental delay; however, detailed phenotyping, treatment history, and longitudinal severity grading for these manifestations were not available from the clinical record at the time of manuscript preparation. Because this is a retrospective single-case report, unavailable historical details are acknowledged as a limitation rather than inferred.

The patient had otherwise been well until she developed progressive fatigue and dyspnea on exertion and was found to be anemic, prompting further evaluation for an occult gastrointestinal source. Available records did not capture the exact duration, symptom frequency, body-weight trajectory, complete physical examination findings, or medication history at the time of presentation. Subsequent workup demonstrated an obstructing tumor in the proximal ascending colon. Baseline cross-sectional imaging showed no metastatic disease. She underwent right hemicolectomy, recovered postoperatively, and was followed according to standard surveillance recommendations; at the time the manuscript was developed, she was also receiving systemic therapy. Because granular inpatient medication administration details, operative approach, and exact treatment dates were not fully available in the source record, we present only verified management information.

## Diagnostics

The results of germline testing, biopsy, immunohistochemistry, imaging, surgical pathology, and tumor sequencing are summarized in Table 2.

Diagnostic evaluation established right-sided colon adenocarcinoma that was mismatch repair deficient and MSI-H. The imaging, endoscopic, and histopathological findings at the time of diagnosis are shown in Figure 2. Colonoscopic biopsy showed adenocarcinoma with isolated loss of *PMS2* and retained expression of *MLH1*, *MSH2*, and *MSH6*. Baseline staging scans showed no metastatic disease. The patient subsequently underwent right hemicolectomy, and surgical pathology identified two foci of invasive adenocarcinoma arising in the ascending colon, measuring 3.5 cm and 4.0 cm, both well differentiated with mucinous features. The lesions were staged as T3 N0 and T2 N0, corresponding to localized stage II disease. Tumor next-generation sequencing demonstrated MSI-H status with a markedly elevated tumor mutational burden of 43 mutations per megabase. Despite the hypermutated MSI-H phenotype, the tumor lacked detectable somatic alterations in *BRAF* or *NF1*.

## Patient perspective

A direct patient statement was not available for inclusion. At the time the manuscript was developed, the patient was undergoing systemic therapy and was unable to participate in a formal perspective interview. We therefore cannot reliably report her views on diagnosis, treatment tolerance, or quality of life beyond the verified clinical record.

## Discussion

This case documents colorectal cancer in a patient with a mosaic monoallelic germline pathogenic *NF1* variant and no identified germline Lynch syndrome-associated variant. Lynch syndrome is the most common hereditary colorectal cancer syndrome and is caused by germline pathogenic variants in DNA mismatch repair genes, most commonly *MLH1*, *MSH2*, *MSH6*, and *PMS2*, or by *EPCAM* deletions affecting *MSH2* expression; it confers increased risk of colorectal and several extracolonic malignancies [20, 21]. A mosaic monoallelic germline pathogenic variant refers to a disease-causing variant present in only a subset of the body’s cells, likely arising after fertilization, with a single altered copy detected in germline-derived tissue at a non-50% allele fraction. In this patient, the saliva VAF of ~35% supports mosaicism rather than constitutional heterozygosity. To our knowledge, this is the first report of colorectal cancer in association with the *NF1* c.1756_1759del (p.Thr586Valfs*18) variant. Prior reports linking *NF1* to colorectal neoplasia remain sparse [16, 17]. Accordingly, this case should be interpreted as hypothesis-generating rather than proof of causality.

A biologic link is plausible because neurofibromin normally restrains RAS–MAPK signaling. The RAS–MAPK pathway transduces extracellular growth signals from membrane receptors to intracellular kinases, ultimately promoting transcriptional programs that regulate proliferation, survival, differentiation, and migration [22, 23]. In colorectal carcinogenesis, dysregulation of this pathway can promote epithelial proliferation, reduce apoptosis, and cooperate with other genomic events during adenoma-to-carcinoma progression [22]. Classic pathway activation occurs through oncogenic alterations in *KRAS*, *NRAS*, or *BRAF*, but loss of negative regulators such as *NF1* may provide an alternative route to pathway activation [24]. Impaired production or function of neurofibromin can therefore contribute to both benign and malignant tumorigenesis in multiple tissues [25]. In the present case, the germline variant is a frameshift predicted to truncate neurofibromin, and prior functional work suggests this recurrent variant markedly reduces neurofibromin expression [26]. However, how this specific variant differs clinically from other loss-of-function *NF1* variants remains uncertain. The currently available evidence does not establish a distinctive colorectal-cancer-prone genotype for c.1756_1759del; rather, the observation raises the possibility that truncating variants in this region may warrant further study.

The tumor phenotype in this case is also notable. dMMR colorectal cancers account for approximately 10% to 15% of localized colorectal cancers and are characterized by accumulation of replication errors at microsatellites, hypermutation, prominent immune infiltration, and often favorable stage-adjusted prognosis in localized disease compared with mismatch repair-proficient tumors [15]. dMMR/MSI-H status is clinically important because it informs Lynch syndrome evaluation and may predict responsiveness to immune checkpoint inhibition in advanced or selected non-metastatic settings [14, 27]. The relationship between *NF1*-related signaling abnormalities and mismatch repair deficiency remains incompletely defined. A reasonable hypothesis is that altered RAS–MAPK signaling may modify the selective landscape in which dMMR clones emerge or expand, but direct mechanistic evidence is lacking in this single case. We therefore frame any putative interaction between *NF1* loss and dMMR/MSI-H biology as speculative and requiring tumor-level and functional validation.

From a clinical-management perspective, usual evaluation of NF1 includes careful physical examination, ophthalmologic assessment, developmental and neurologic review, and targeted imaging when symptoms suggest internal tumors or other complications [28, 29]. Management is individualized and often multidisciplinary, focusing on surveillance and treatment of complications rather than prophylactic cancer-directed intervention. Standard workup for colon cancer includes history and examination, laboratory assessment including evaluation for anemia, colonoscopy with biopsy, staging imaging, pathologic staging, and universal mismatch repair testing [30]. Standard treatment for localized right-sided colon adenocarcinoma commonly includes surgical resection. Hemicolectomy is resection of one side of the colon; right hemicolectomy removes the cecum, ascending colon, hepatic flexure, and variable terminal ileum with regional lymphovascular pedicle. It is indicated for malignant neoplasms of the right colon and selected benign conditions, while contraindications are relative and depend on operative risk, extent of disease, and patient physiology. Potential risks include bleeding, infection, anastomotic leak, ileus, bowel dysfunction, thromboembolism, and, in some cases, need for diversion or reoperation. For stage II colon cancer, prognosis is generally favorable after complete resection, although risk stratification depends on pathologic features and molecular context. Localized dMMR/MSI-H tumors often have relatively favorable outcomes, whereas adverse molecular co-alterations can modify prognosis [31, 32]. The specific systemic regimen used in this patient was not fully documented in the available source material; accordingly, we do not speculate on drug class, dosage, or toxicity profile beyond noting that systemic therapy was ongoing during manuscript preparation.

This case has important limitations. First, it represents a single observation and cannot establish causality or estimate risk. Second, detailed longitudinal clinical data, including symptom timing, medication doses, operative approach, and patient-reported outcomes, were incompletely available because of the retrospective nature of the report. Third, tumor-level *NF1* analysis was limited, and functional validation was not performed; therefore, biallelic inactivation of *NF1* in tumor tissue was not demonstrated. These limitations are central when interpreting any proposed biological relationship between mosaic *NF1* variation and colorectal carcinogenesis. Even so, the case expands the phenotypic context in which pathogenic *NF1* variants may be considered and supports future genotype-first studies incorporating paired germline-tumor sequencing, tumor-level copy-number and expression analyses, and functional studies of candidate variants.

## Conclusions

This case underscores the need for systematic tumor-level analyses and larger genotype-first cohorts to clarify whether mosaic or incidentally identified pathogenic *NF1* variants contribute to colorectal cancer risk. It also highlights the importance of universal mismatch repair testing and careful interpretation of rare genotype-phenotype observations as hypothesis-generating signals that may inform future personalized surveillance research [1, 6].

## Figures and Tables

**Figure 1.**
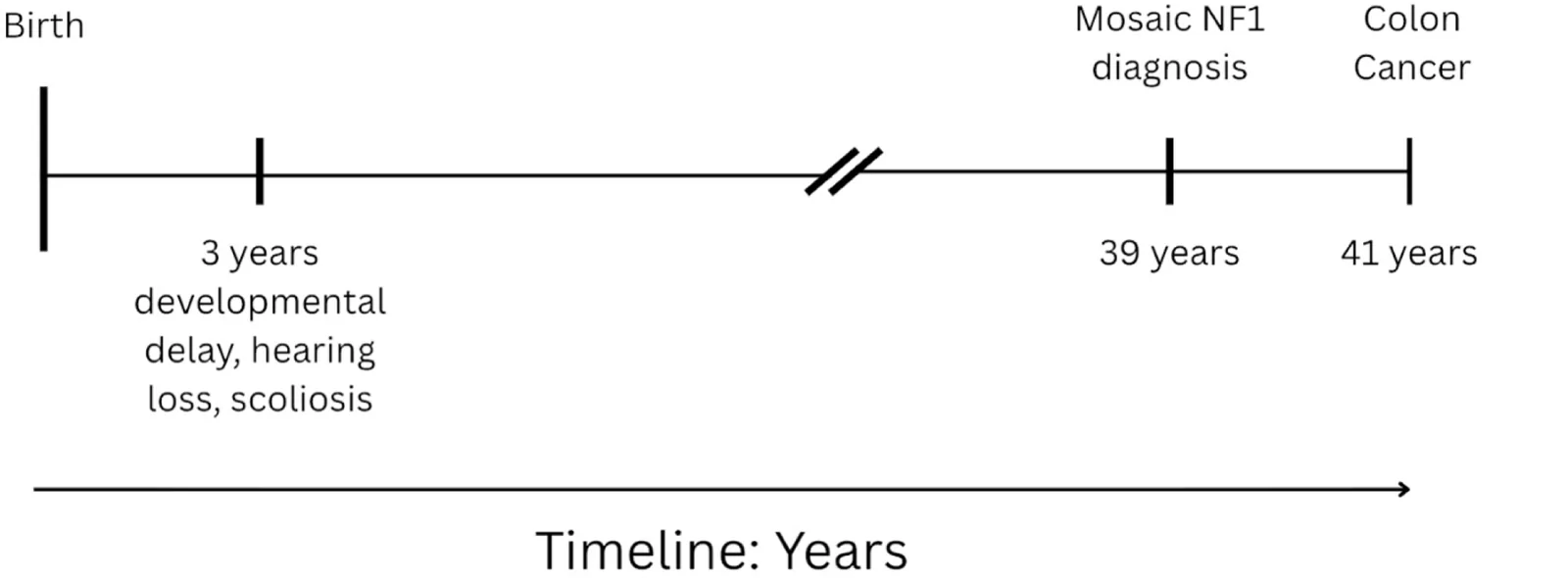
Clinical timeline illustrating the sequential presentation of key diagnoses and comorbidities. NF1: neurofibromatosis type 1.

**Figure 2.**
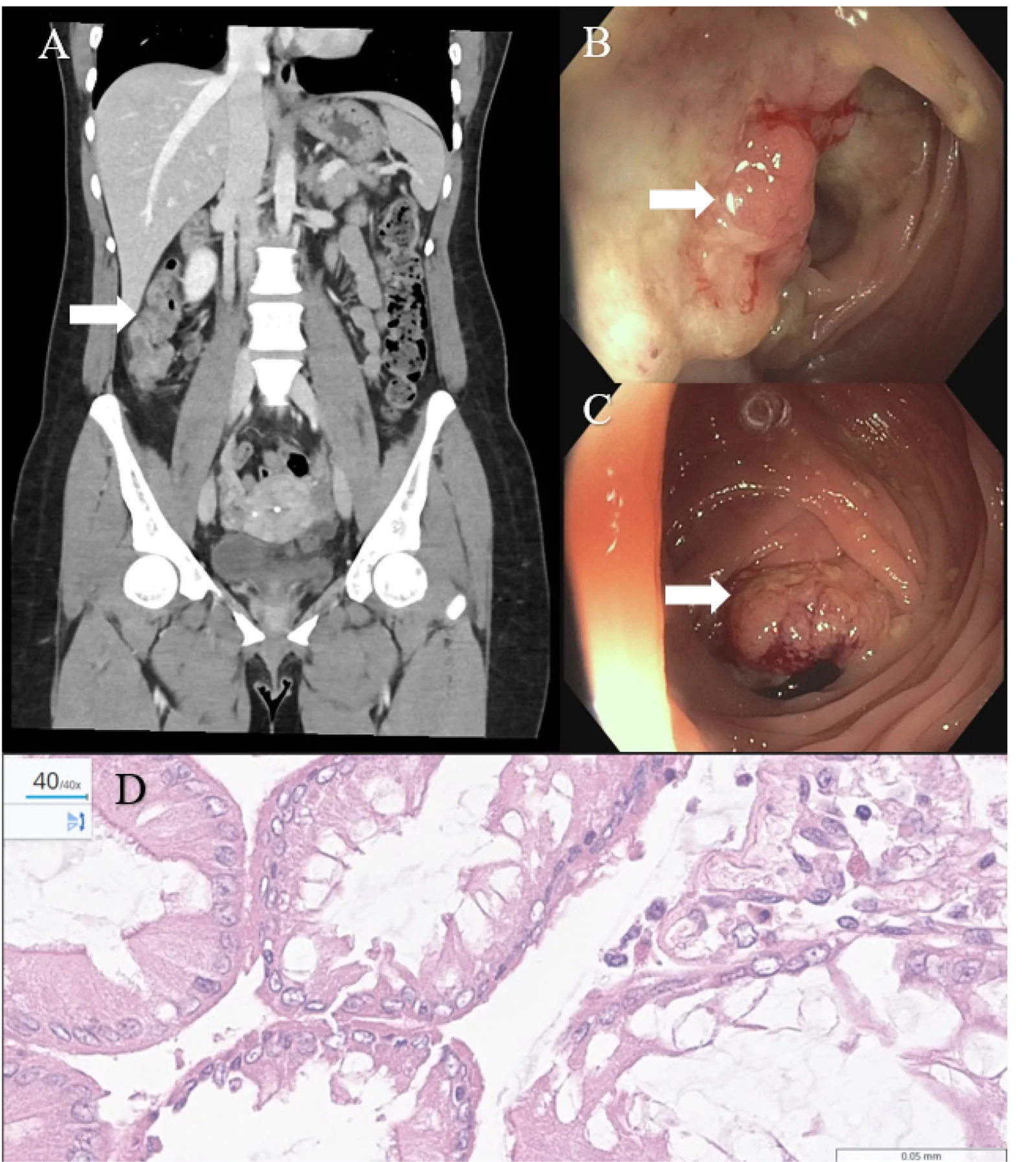
Diagnostic Evaluation of Ascending Colon Adenocarcinoma. (**A**) Coronal CT abdomen and pelvis with intravenous contrast showing a long segment of ascending colon demonstrating mild wall thickening and adjacent fat stranding concerning for an underlying structural process (white arrow). (**B, C**) Colonoscopy showing a frond-like/villous and ulcerated partially obstructing large circumferential mass (white arrows) in the proximal ascending colon. (**D**) Biopsies and pathological examination [including hematoxylin and eosin (HE) staining] showed invasive colorectal adenocarcinoma.

**Table 1.** Clinical timeline of diagnostic workup, treatment, and follow-up.

Clinical period	Key events	Verified findings/management
Approximately 2 years before colon cancer diagnosis	Germline multigene testing performed because of hearing loss, scoliosis, and developmental delay	Mosaic monoallelic pathogenic *NF1* variant NM_000267.3:c.1756_1759del (p.Thr586Valfs*18) identified in saliva at variant allele frequency (VAF) ~35%; Lynch syndrome-associated germline variants not detected
Presentation period	Evaluation of fatigue, dyspnea on exertion, and anemia	Symptoms prompted gastrointestinal workup for occult blood loss and structural lesion
Diagnostic workup	Imaging, colonoscopy, biopsy, mismatch repair immunohistochemistry, and tumor sequencing	Obstructing proximal ascending colon tumor identified; biopsy confirmed adenocarcinoma; dMMR/MSI-H tumor profile established
Definitive local treatment	Right hemicolectomy	Two foci of invasive adenocarcinoma (3.5 cm and 4.0 cm), well differentiated with mucinous features; staged as T3 N0 and T2 N0 (stage II)
Postoperative course and follow-up	Recovery and surveillance	Recovered postoperatively and followed per standard surveillance protocol; systemic therapy was ongoing at manuscript development

**Table 2.** Summary of diagnostic tests, verified results, and clinical interpretation.

Test category	Verified result	Interpretation
Germline multigene panel (saliva)	Mosaic monoallelic pathogenic *NF1* variant NM_000267.3:c.1756_1759del (p.Thr586Valfs*18); variant allele frequency (VAF) ~35%; no pathogenic Lynch syndrome-associated germline variant detected	Supports post-zygotic mosaic NF1 and argues against an inherited mismatch repair syndrome identified on the germline panel
Biopsy pathology	Adenocarcinoma	Established malignant epithelial tumor of colonic origin
Mismatch repair immunohistochemistry	Isolated loss of postmeiotic segregation increased 2 (*PMS2*); retained mutL homolog 1 (*MLH1*), mutS homolog 2 (*MSH2*), and mutS homolog 6 (*MSH6*)	Pattern consistent with mismatch repair deficiency; prompted molecular characterization and Lynch syndrome consideration
Baseline imaging	No metastatic disease identified	Supported localized disease at diagnosis
Surgical pathology	Two foci of invasive adenocarcinoma (3.5 cm and 4.0 cm) in the ascending colon, well differentiated with mucinous features; T3 N0 and T2 N0	Localized stage II colon adenocarcinoma. T3 N0 indicates tumor extension through the muscularis propria into pericolonic tissues without regional nodal metastasis; T2 N0 indicates invasion into, but not through, the muscularis propria without nodal metastasis
Tumor next-generation sequencing	Microsatellite instability-high (MSI-H); tumor mutational burden 43 mutations/Mb; no somatic *BRAF* or *NF1* alteration detected	Hypermutated dMMR tumor profile with no detected somatic *BRAF* driver or second somatic *NF1* event in available testing

dMMR: deficient mismatch repair; NF1: neurofibromatosis type 1.

## Data Availability

The data supporting the findings of this case report are available from the corresponding author upon reasonable request.

## Abbreviations

dMMR: deficient mismatch repair
MAPK: mitogen-activated protein kinase
*MLH1*: mutL homolog 1
*MSH2*: mutS homolog 2
MSI-H: microsatellite instability-high
NF1: neurofibromatosis type 1
*PMS2*: postmeiotic segregation increased 2
RAS: rat sarcoma
VAF: variant allele frequency
